# Research note: *In vitro* anticoccidial activity of protein and lipid extracts from the black soldier fly larvae (*Hermetia illucens*)

**DOI:** 10.1016/j.psj.2025.105009

**Published:** 2025-03-08

**Authors:** Laura Sedano, Maryline Abert Vian, Côme Guidou, Francoise I. Bussière, Sonia Lacroix-Lamandé, Christophe Trespeuch, Bertrand Méda, Anne Silvestre

**Affiliations:** a INRAE, UMR ISP, Université de Tours, Nouzilly 37380, France; b INRAE, UMR SQPOV, Green Team, Avignon Université, Avignon F-84000, France; c MUTATEC, Cavaillon 84300, France; d INRAE, BOA, Université de Tours, Nouzilly 37380, France

**Keywords:** Chicken, Poultry, *Eimeria tenella*, Coccidiosis, Insect

## Abstract

Avian coccidiosis, caused by *Eimeria spp*., is the main parasitic disease in the poultry industry, responsible for high economic costs worldwide. Faced with anticoccidial resistance and societal pressure to reduce inputs in livestock sector, insects could provide a relevant alternative to anticoccidial molecules. The larvae of the black soldier fly (*Hermetia illucens*) are easy to rear, and can be used to enhance the value of by-products and food waste. Here, anticoccidial activities of protein extracts solubilized in water and lipid extracts solubilized in methanol of *H. illucens* larvae were evaluated *in vitro*. Larvae were either blanched and freeze-dried or dried and pressed. The maximum noncytotoxic dose of each extract (20 g/L and 35 g/L of dry matter equivalent) was assessed in avian cells, using a series of tenfold dilutions. The parasite strain *Et*-INRAE was modified to express nano-luciferase reporter gene. Parasites were pre-treated with extracts. Then, avian cells were infected and incubated in the presence of the extracts. Inhibition of cell invasion and parasite development were assessed by quantification of the luminescence detected. Lipid extracts and protein extracts inhibit *Eimeria* growth at, at least, a 10⁻⁶ dilution. Further research is required to confirm these results *in vivo*, assess potential antinutritional effects, and possibly identify active compounds from fractionated extracts to optimize the observed anticoccidial activities.

## Introduction

Avian coccidiosis is a cosmopolitan disease and represents a significant economic threat to the poultry industry . The disease is caused by various species of *Eimeria*, an obligate intracellular parasite that infects the digestive tract of their hosts. In a recent update, ten avian *Eimeria* species were described, among which *E. tenella, E. necatrix, E. brunetti, E. maxima, E. acervulina* are economically significant (Blake, 2025). The life cycle of *Eimeria sp*. occurs within a single host and includes both asexual and sexual phases, which produce large quantities of oocysts, excreted by the infected animal into the environment. Prophylaxis relies on the use of vaccines to stimulate protective immunity and on anticoccidial feed additives to inhibit parasite development. Only a limited number of anticoccidial molecules are available (synthetic or ionophores molecules), and most of them encounter resistance development in field populations of parasites. The increasing emphasis on reducing antibiotic and antiparasitic treatments makes controlling certain diseases more challenging, highlighting the need for alternative and effective prevention methods.

Since insects are a natural component of the diet of birds, numerous studies and reviews have been published over the last decade on the various *in vivo* positive effects of using *Hermetia illucens* larvae, the black soldier fly (BSF), to feed poultry (for a recent review, e.g. Slimen et al., 2023). These studies reported various *in vivo* benefits on animal performance, meat quality or animal welfare. Consequently, the use of live insects, lipid extracts, and more recently, processed animal proteins derived from insects (insect PAPs) is now authorized for feed poultry within the European Union (Commission Regulation, EU 2021/1372). The production of insects for animal feed is experiencing significant development to improve protein self-sufficiency and animal welfare through environmental enrichment. In particular, the larvae of BSF are easy to rear and can upcycle a large variety of by-products and food waste. In addition, insect larvae also contain compounds, such as chitin, lauric acid, antioxidant compounds and antimicrobial peptides (AMPs), that are beneficial for gut microbiota and animal health (Slimen et al., 2023). However, the potential of insects for their antiparasitic effects has been very little investigated.

In the present study, we evaluated the anticoccidial activities of various BSF extracts, against asexual multiplication of *Eimeria tenella* (one of the most pathogenic species, responsible for caecal coccidiosis in chickens). The early asexual stages can be reproduced *in vitro*. We developed recombinant *E. tenella* strains that express luciferase, allowing the quantification of invasion and intracellular development. We performed a cytotoxicity assay to determine the highest non-cytotoxic concentration for each extract, and evaluated their antiparasitic activities.

## Material and methods

All animal experimentations have been performed in the Infectiology of Farm, Model, and Wildlife Animals Facility (PFIE, Centre INRAE Val De Loire; https://doi.org/10.15454/1.5572352821559333E12). Experimental protocols were designed in compliance with French law (2010/63/EU, 2010; Rural Code, 2018; Decree No. 2013-118, 2013) concerning the use of laboratory animals. Care and euthanasia of animals were practiced according to the national ethical guidelines and approved by the local ethics committee for animal experimentation (Comité d'Ethique en Expérimentation Animale Val de Loire, CEA VdL N°19): APAFIS#25884.

### Cell culture and parasite strains

*Eimeria tenella* parasites were propagated in outbred PA12 chickens (4-6 weeks old), orally infected with 10^4^ sporulated oocysts and reared in a coccidia-free environment with an *ad libitum* supply of filtered water and anticoccidial- and antibiotic-free feed. The parental strain *Et*-INRAE was modified (Ribeiro E Silva et al., 2021) to express nano-luciferase reporter gene, according to the protocol previously described by Clark et al. (2008). Briefly, we generated the recombinant strain expressing either the nano-luciferase in fusion with YFP, under *E. tenella* actin 5′UTR (for invasion assay) or the nano-luciferase in fusion with mcherry, under *E. tenella* SAG (surface antigen) 5′UTR (for development assay). Recombinant parasites were obtained by electroporation of parental *Et-*INRAE sporozoites and inoculated to chicken cloaca. Seven days post-infection, the chickens were euthanized, and parasites were recovered from the ceca. Sporulated oocysts were sorted by fluorescence-activated cell sorting to select recombinant oocysts that were successively propagated in chickens to stabilize recombinant populations of parasites. The parental and the modified strains were susceptible to coccidiostats.

Unsporulated oocysts were collected from caeca and purified. Sporulated oocysts were obtained after an incubation in a water bath under agitation at 27°C during 72 h in a solution of potassium dichromate 2.5 % (Sigma-Aldrich, St-Louis, USA). Sporozoites were purified from sporulated oocysts: oocyst walls were broken with 0.5 mm glass beads and sporocysts were incubated in 0.25 % trypsin solution from porcine pancreas (Sigma-Aldrich, St-Louis, USA), 0.5 % porcine biliary salts (Sigma-Aldrich, St-Louis, USA) in PBS, pH 7.4 at 41°C for 1h. Finally, sporozoites were purified by a filtration on cotton followed by a filtration on polycarbonate filters (5 µM; Whatman, Florham Park, USA). CLEC213, a chicken lung epithelial cell line (Esnault et al., 2011), were grown in DMEM/F-12 (Gibco; Life Technologies Limited, Paisley, UK), 10 % of Fetal Bovine Serum (Dutscher, Bernolsheim, France) and 1 % of penicillin-streptomycin (Cytiva, Hyclone, Laboratories, South Logan, USA) at 41°C, 5 % CO2. For all *in vitro* assays, cells were harvested with 0.05 % trypsin-EDTA (Gibco; Life Technologies Limited, Paisley, UK) and 1.5 × 10^4^ cells were allowed to adhere overnight in 96-wells plate.

### BSF extracts production

**Fig. 1:**
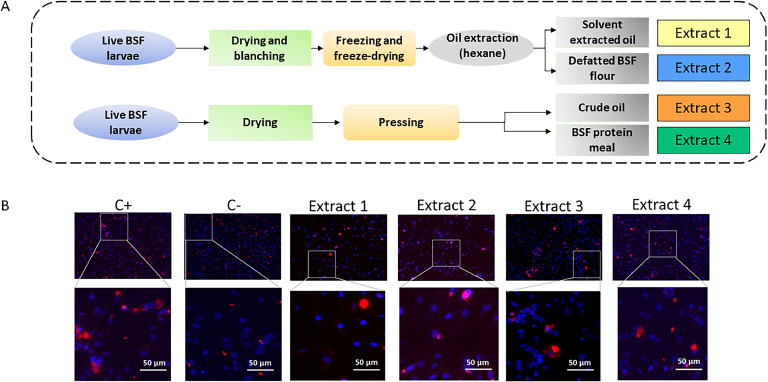
Lipid and protein extracts preparation from live black soldier fly (BSF) larvae (A). Lipids were dissolved in hexane for extraction, while proteins were extracted in water. (B) Immunofluorescent assay performed on avian cells infected by *E. tenella* parasites, incubated with lipid extracts 1 and 3 (10^-3^ dilution factor) or with protein extracts 2 and 4 (10^-5^ dilution factor), 72 h post-infection. The positive control (C+) represents the condition using parasites incubated without extracts, while the negative control (C-) represents the condition using heat-inactivated parasites.

The live BSF larvae were produced from MUTATEC (Cavaillon, France), a French insect producer. The larvae were mainly reared on waste fruits and vegetables. We compared protein extracts solubilized in water and lipid extracts solubilized in methanol (Fig. 1A) from BSF larvae that were: either blanched and freeze-dried (Extracts 1 and 2), or dried and pressed (Extracts 3 and 4). Two batches of extracts were produced: the first batch was used as is, and the second was sterilized by filtration (0.2 µm). The extracts of batch 1 and 2 were prepared at a concentration of 20 g/L and 35 g/L of dry matter equivalent, respectively. For each extract, a series of tenfold dilutions was prepared, ranging from 10 ^to 1^ to 10^-6^.

### Cytotoxicity assay

To determine the maximal noncytotoxic concentration, extracts were incubated with CLEC213 cells at different concentrations for 72 h at 41°C. Then, culture medium was removed and replaced by a fresh one. To quantify cytotoxicity, MTS was added following manufactory's instructions (CellTiter 96® Assay; Promega, Madison, USA) and cells were incubated at 37°C for 4h. Absorbance was read at 492 nm on a microplate reader (Thermo Multiskan Ascent; Thermo Fisher Scientific, Waltham, USA). The relative cell viability was calculated as the percentage of untreated cells. Data were analysed according to the ISO-10993-5 standard and >80 % of cell viability was chosen as cut off.

### *In vitro* anticoccidial assay

The highest non-cytotoxic dose of extract (i.e. maintaining cell viability above 80 %) was used to assess its effect on cell invasion by sporozoites and intracellular parasite development. First, 1.5 × 10^4^ sporozoites (MOI 1) were incubated with extract at different concentration in culture medium, during 1 h at room temperature. For controls, 1.5 × 10^4^ parasites were incubated for 1 h at room temperature without compound as an untreated positive control (C+, set up at 100 % invasion) while the negative control (C-) was prepared with 1.5 × 10^4^ parasites incubated for 1 h at 60°C without compound (heat-killed sporozoites control). The medium from overnight-adherent CLEC213 cells was removed, and the cells were infected with the sporozoite suspensions for 2 h at 41°C to evaluate invasion, and for 72 h to assess development. After incubation, wells were washed three times with PBS with a microplate washer (Hydroflex; Tecan, Männedorf, Switzerland) to eliminate the extracellular parasites. To quantify sporozoite invasion and development, 20 µL of a mix of Nano-Glo® Luciferase Assay Substrate and Nano-Glo® Luciferase Assay Buffer was added to wells, following manufacturer's instructions (Nano-Glo® Luciferase Assay System; Promega, Madison, USA) and luminescence was measured in a Glo-Max®-Multi Detection System (Promega, Madison, USA). Extract effect on CLEC213 cells invasion and parasite development were established as the percentage of untreated sporozoites control (C+).

### Indirect immunofluorescent assay

This assay was performed in the same conditions as for the *in vitro* anticoccidial assay, except it was performed with 2 × 10^5^ CLEC213 cells in 24-well plates, at MOI = 1. Infected cells were washed at 2 h or at 72 h post infection, for the invasion assay and the development assay, respectively. Cells were fixed in 2.7 % paraformaldehyde (PFA). Monolayers were mounted in Vectashield mounting medium containing 1.5 µg/ml of DAPI (4=,6-diamidino-2- phenylindole; Clinisciences, France) to label the nuclei. Parasite staining was performed with a polyclonal anti-Eimeria spp. antibody (1:500) produced in our lab and revealed by an Alexa 594-conjugated secondary antibody (Life Technologies, no. A11012). Infected cells were examined by fluorescence microscopy (Zeiss Axiovert 200 microscope; Carl Zeiss, Germany).

### Statistical analysis

For cytotoxicity assay and for anticoccidial activity, 9 wells from 3 independent experiments were analysed. The differences between results obtained in treated and untreated conditions were expressed in percent of the positive control values. The results were expressed as means ± SEM. A Kruskal–Wallis test with Dunn's test was used to compare results from cells treated with different concentrations of plant extracts with results from control cells, using GraphPad Prism v8.4.3 (GraphPad Software Inc., La Jolla, USA). Statistical significance levels were considered as follows: *p*-value < 0.05 (*), *p*-value < 0.01 (**), *p*-value < 0.001 (***) and *p*-value < 0.0001 (****).

## Results and discussion

In our *in vitro* avian cell model, regardless of the solvent used, the 10⁻¹ dilution was cytotoxic and was therefore excluded from the cell invasion and development inhibition assays. For all other dilutions, no cytotoxicity was observed when compounds were incubated for 2 h; only a moderate cytotoxicity was observed after 72 h incubation of extract 1 and extract 3 at dilution 10^-2^. Extract 1 and 3 are lipid extracts solubilized in methanol, whose toxicity towards cells is known (Tanneberger et al., 2010).

The indirect immunofluorescent assay (IFA, Fig. 1B) showed that in the positive control, where no extract is added, the sporozoite parasite (small red signal) developed into schizont (larger red signal), whereas, in the negative control, parasites were heat-killed, and only sporozoites are detected, supporting the absence of development. When extracts were added, a part of the parasite stay as sporozoites stage, which means that they were killed by the extracts and did not invade or develop into schizont. To quantify the anticoccidial effect of extracts, we used a *E. tenella* recombinant strain expressing the nano-luciferase as reporter gene.

**Fig. 2:**
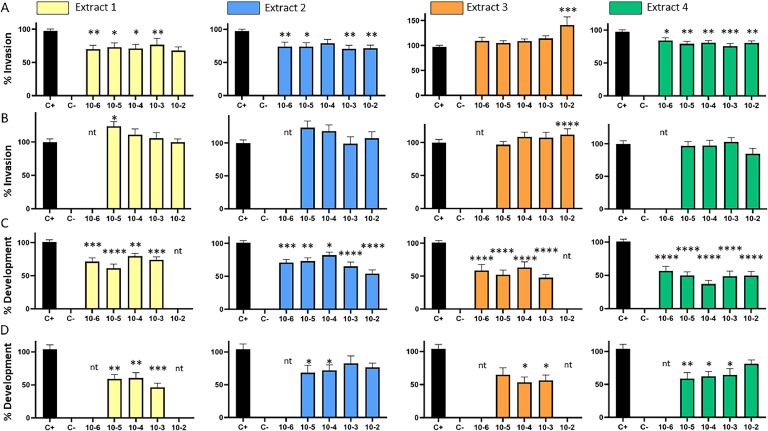
Effect of the four extracts on the invasion of CLEC213 cells (A, B) and the development (C, D) of *E. tenella* parasites. A and C: Batch 1 prepared at 20 g/L of dry matter equivalent (unsterilized). B and D: Batch 2 prepared at 35 g/L of dry matter equivalent sterilized by filtration (0.2 µm). The positive control (C+) represents the condition using parasites incubated without extracts, while the negative control (C-) represents the condition using heat-inactivated parasites. nt: not tested.

Although a moderate inhibition of invasion (Fig. 2A) was observed with the 4 native extracts (batch 1), the effect was no longer observed after their sterilization (batch 2; Fig. 2B). It can be assumed that sterilization by 0.2 µm filtration eliminated or reduced the quantity of active compounds.

The best inhibition results were achieved at 72h.p.i., which assesses the cumulative effects of the extracts on the extracellular parasite viability, host cell invasion inhibition, and intracellular development inhibition (Fig. 2C and D). Protein extract 4 was subjected to heat treatment for drying the larvae, in contrast to protein extract 2. The protein extracts inhibit the development of *Eimeria* at 10^-6^ dilutions. The lipid extracts seem as effective, with activity observed at a 10⁻⁶ dilution. No dose-dependent effect was observed, making it impossible to estimate the 50 % inhibitory concentration.

These *in vitro* results allow us to quantify the effects of the extracts (protein vs*.* lipid) on the different stages of the parasite cycle, both extra- and intracellular. After sterilization by filtration of the extracts, invasion inhibition was no longer observed, whereas, development inhibition was less efficient but still detected. This indicates that active compounds present in the extracts are involved in the anticoccidial activity: some active compounds have a direct antiparasitic activity on the extracellular parasite; other compounds, still present after filtration, help to limit parasite development. Further study of various candidates could be achieved by combining extract fractionation, detailed characterization and evaluation of their anticoccidial activity *in vitro*. In particular, the effects of defensins (Guyot et al., 2020), chitin, lauric acid as well as antioxidant compounds (Mouithys-Mickalad et al., 2020) have been demonstrated in poultry. In a longer-term perspective, the selection of substrates on which BSF larvae are fed could help to direct the composition of AMPs and active compounds of interest.

In conclusion, both the native protein extracts and the lipid extracts exhibited anticoccidial activity, under our conditions. Further research is required to identify and isolate for each type of extract, the compounds responsible for these effects (Slimen et al., 2023) and to validate the promising results under conditions more representative of *in vivo* environments (Osuch et al., 2024). In such contexts, it will be particularly crucial to ensure that the observed anticoccidial activities do not negatively impact zootechnical parameters, as some active compounds may exhibit antinutritional properties at high doses (Gasco et al., 2021).

## Disclosures

The authors declare no conflict of interest. The funders had no role in the design of the study; in the collection, analyses, or interpretation of data; in the writing of the manuscript, or in the decision to publish the results.

## Declaration of competing interest

The authors declare no conflict of interest. The funders had no role in the design of the study; in the collection, analyses, or interpretation of data; in the writing of the manuscript, or in the decision to publish the results.
